# Energy Dispersive Spectroscopy of Open Dentinal Tubules on Application of Nanocrystalline Hydroxyapatite: An In Vitro Study

**DOI:** 10.12688/f1000research.173988.1

**Published:** 2026-05-12

**Authors:** Tripti Rawat, Rashmi Srinath, N C Praveen, B S Amisha, Inderjit Murugendrappa Gowdar, Sultan A. Almalki, Khalid Gufran, Usha G V, Bhuvaneshwari Nadar

**Affiliations:** 1 Periodontics, Clove Dental, Plot No -107, Pocket - 27, Rohini Sector 24,, New Delhi, 110085, India; 2 Department of Periodontics, College of Dental Sciences, Davangere, Karnataka, 577004, India; 3 Periodontics, Clove Dental, 8th main , 11th Cross, Wilson garden Bengaluru, Karnataka, 560027, India; 4 Department of Preventive Dental Sciences,College of Dentistry, Prince Sattam bin Abdulaziz University, Al Kharj, Riyadh Province, 11942, Saudi Arabia; 5 Department of Public Health Dentistry, Bapuji Dental College and Hospital, Davangere, Karnataka, 577004, India; 6 Public Health Dentistry, Terna Dental College and Hospital, Navi Mumbai, Maharashtra, 400706, India

**Keywords:** Dentin Hypersensitivity; Diode laser; Scanning electron microscope; nanocrystalline hydroxyapatite

## Abstract

**Background:**

Dentinal hypersensitivity is an enigmatic problem that has long bewildered the dental profession and is one domain where man has not yet achieved the elusive “gold standards” of management. Recently, lasers have been used owning to their various modes of action to reduce hypersensitivity. Thus, the aim of the present in vitro study is to evaluate and compare the effect diode laser along with and without Nanocrystalline Hydroxyapatite powder on occlusion of dentinal tubules under scanning electron microscope (SEM).

**Methodology:**

Twenty specimens were obtained from dentin discs of 2 mm thickness prepared from the cemento-enamel junction (CEJ) portion of 20 extracted human third molar teeth. The specimens were divided in to four groups of 5 specimens each. The specimens were etched in 37% phosphoric acid for 60 seconds to simulate hypersensitivity condition. Specimens in Group 1 (control) received no further treatment. Group 2 (DL) specimens received irradiation with 810 nm diode laser at an output power of 1 W for 60 seconds in continuous wave, non-contact mode. Specimens in Group 3 received treatment with a single application of nanocrystalline hydroxyapatite powder alone, whereas, Group 4 received a combination treatment of Diode laser irradiation followed by application of nanocrystalline hydroxyapatite powder.

**Results:**

The average tubular diameter of dentinal tubules observed in the specimens belonging to Group 1 (control) was 3.14 microns, and in the Group 2 (diode laser) the average dentinal tubular diameter to be 2.28 microns. Mean diameter of dentinal tubules in Group 3 (nanoHA alone) was 2.41 microns. The specimens belonging to Group 4 (DL + nanoHA) showed average dentinal tubular diameter were 1.74 microns.

**Conclusion:**

The single application of nanocrystalline hydroxyapatite powder and diode laser irradiation, showed significantly greater tubular occlusion and increased reduction in dentinal tubular diameters showing greater potential in occluding open dentinal tubules when compared to diode laser alone.

## Introduction

Dentin hypersensitivity is indeed a prevalent issue in dental practice, defined as pain resulting from exposed dentin reacting to various stimuli—chemical, thermal, tactile, or osmotic—without any underlying dental pathology. ^1^ The exposure of dentinal tubules to the oral environment can occur due to several factors, including parafunctional habits (such as bruxism), improper tooth brushing techniques, excessive consumption of acidic beverages, and frequent use of certain mouthwashes. ^2^ This exposure can lead to both physiological and psychological discomfort, significantly impacting an individual’s oral health-related quality of life. The wide range in prevalence reported in a recent study between 1.3% and 91% highlights the variability in individual experiences. ^3^

Dentin hypersensitivity indeed develops in two phases, with the first phase being lesion localization. During this phase, various factors—such as attrition, abrasion, erosion, and gingival recession—result in the loss of enamel and cementum. This loss exposes the underlying dentin, which can lead to a thinning of the calcified smear layer that normally provides some protection to the dentinal tubules. As the protective layers are compromised, the dentin becomes more susceptible to external stimuli, leading to increased fluid movement within the dentinal tubules. This fluid movement activates the sensory nerves, resulting in the characteristic pain associated with dentin hypersensitivity. ^4^ The second phase of dentin hypersensitivity, known as lesion initiation, occurs when the protective barriers such as tubular plugs and the smear layer are removed. As these protective layers are compromised, the dentinal tubules become fully exposed to the external environment. This exposure allows external stimuli to directly affect the dentin and pulp. The heightened sensitivity in this phase is due to the direct stimulation of the nerve endings within the dentin and the pulp, leading to significant discomfort or pain. ^5^

The findings from scanning electron microscopy and dye penetration studies indicate that hypersensitive dentin has a significantly higher number of tubules and larger diameters compared to non-sensitive dentin. Specifically, hypersensitive dentin features eight times more tubules and tubules that are twice the diameter of those in non-sensitive dentin. The increased tubule diameter is particularly noteworthy, as fluid flow through these tubules is proportional to the fourth power of the radius. This means that even a small increase in diameter can lead to a substantial rise in fluid flow—doubling the diameter results in a 16-fold increase in fluid movement. ^6^

The management of dentinal hypersensitivity focuses on alleviating pain or discomfort through two primary strategies such as occluding agents and direct ionic diffusion. ^7^ Various laser types have been tested for DH treatment, including Neodymium- or Erbium-doped yttrium-aluminum garnet (Nd:YAG and Er:YAG), CO _2_, He-Ne, and diode (ie, GaAlAs) lasers, with various energy settings and with wavelengths ranging from 632.8 nm (He-Ne) to 10,600 nm (Er:YAG, CO _2_). The mechanism of laser effects on dentin hypersensitivity is thought to be the laser- induced occlusion or narrowing of dentinal tubules as well as direct nerve analgesia. ^8^ They cause occluding of tubules through coagulation of proteins which diminishes the fluid movement, causing partial sub-melting of the denuded dentin and discharging of the internal tubular nerve.

Diode lasers have emerged as a promising option for treating dentinal hypersensitivity due to their specific parameters, which result in fewer damaging changes to the root surface and minimal temperature increases in the irradiated area. ^9^ This makes them a safer and more efficient choice compared to other laser types. Given that microstructural changes in dentinal tubules are not clearly observable in in-vivo studies, in vitro research becomes essential. Scanning electron microscopy (SEM) is used in these studies to closely examine the occlusion of dentinal tubules under high magnification, providing detailed insights into the effectiveness of different treatment modalities. ^10, 11^ The aim of the present study is to evaluate the efficacy of diode laser treatment, both with and without the application of nanocrystalline hydroxyapatite crystals, in occluding dentinal tubules. By comparing these two approaches, the study seeks to determine whether the addition of hydroxyapatite enhances the occlusive effects of the diode laser, thus improving outcomes for patients with dentinal hypersensitivity. The findings from this research could provide valuable guidance for clinical practices aimed at managing this condition effectively.

## Materials and methods

The present invitro study, adhered to the Declaration of Helsinki and was approved by the Institutional Ethical Committee, College of Dental Sciences, Davangere (Approval No: CODS/263/2020–2021). Written informed consent was obtained from all participants for use of extracted teeth and for publication of data. Twenty extracted human third molars were included in the study. Teeth presenting carious lesions, intrinsic stains, restorations, or abnormal morphology were excluded. From the coronal portion above the cemento-enamel junction, dentin discs of 2 mm thickness were prepared using diamond cylindrical burs with a high-speed handpiece. ^12^

The discs were sequentially polished with silicon carbide papers of 400, 600, 800, and 1000 grit to produce a standardized smear layer. Each disc was sectioned into four quadrants using a diamond disc bur, and the quadrants were randomly allocated to four groups, ensuring that specimens for both control and test groups originated from the same tooth. ^13^ The groups were defined as follows: Group 1 – Control (no treatment), Group 2 – Diode laser, Group 3 – Nanocrystalline Hydroxyapatite (Sigma Aldrich, USA), and Group 4 – Diode laser combined with Nanocrystalline Hydroxyapatite. Following allocation, specimens were mounted on glass slides with cyanoacrylate adhesive and ultrasonicated in distilled water for two minutes to eliminate residual smear layer. ^12^

The specimens were etched with 37% phosphoric acid for 60 s to simulate hypersensitivity conditions ^14^ and stored in artificial saliva throughout the experimental period. ^15^ Specimens in Groups 2 and 4 were irradiated twice for 5 s using a diode laser (810 nm, 1 W) [Picasso, AMD lasers, USA] delivered through a 300 μm optic fiber attached to a straight handpiece. The laser tip was positioned perpendicular to the dentin surface at a 1 mm distance to avoid contamination. Following treatment, discs were dehydrated at 70 °C for 24 h, mounted on stubs with double-faced carbon tape, sputter-coated with a 30 μm gold-platinum layer in a vacuum apparatus, and examined under SEM.

### SEM analysis

**Figure 1.**
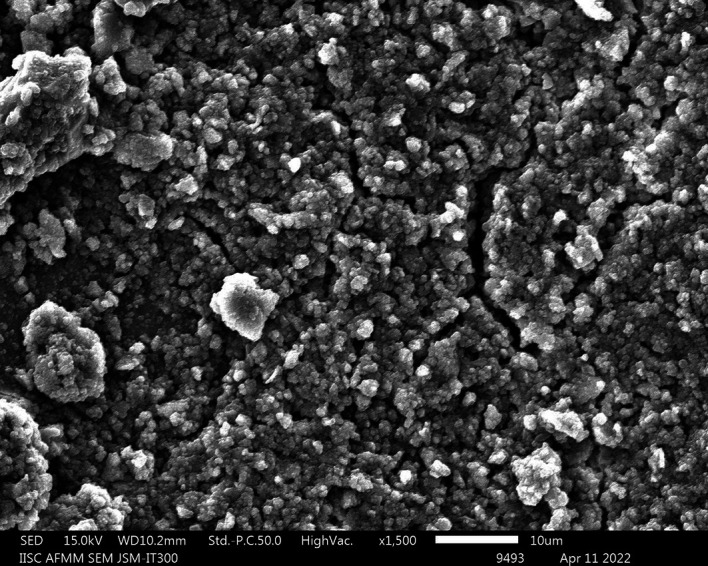
SEM micrograph showing diameter of dentinal tubules.

**Figure 2.**
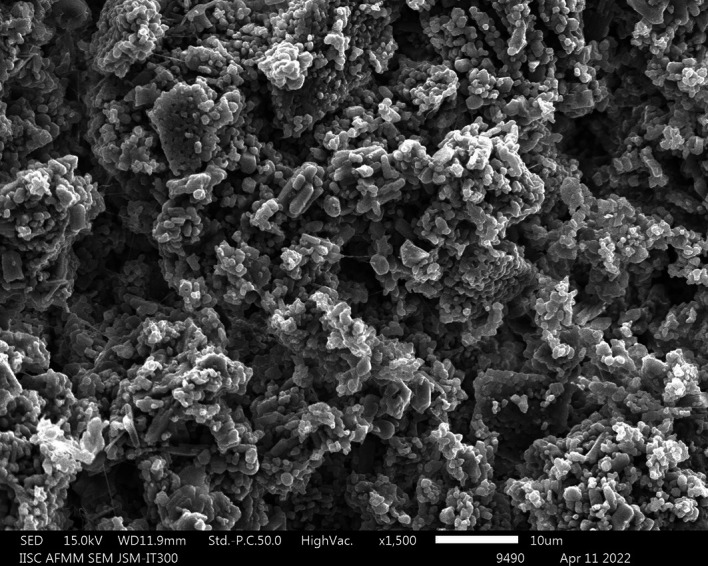
Surface topography of dentin demonstrating tubule occlusion.

Specimens were fixed in 2.5% glutaraldehyde (0.1 M phosphate buffer, pH 7.4) for 24 h, washed, and dehydrated through graded alcohols before mounting on SEM stubs. After air-drying (48 h), they were sputter-coated with 30–40 nm gold (BAL-TEC SCD-500) and examined under SEM (JEOL JSM-IT300, 15 kV). Tubular diameter and occlusion were assessed by scanning central areas to obtain circular tubule cross-sections, ^12^ and representative photomicrographs were captured at 1500× ( Figures 1 and 2).

**Calculation of number of tubules**

Tubules were manually counted in photomicrographs, and the total number per sq. mm was calculated using the formula ^16^:

Total number of tubules per sq.mm = 1000000 × N/area of the photomicrograph in sq. microns. (‘N’ being the number of tubules observed in the specimen).

**Measurement of diameter of tubules**

**Figure 3.**
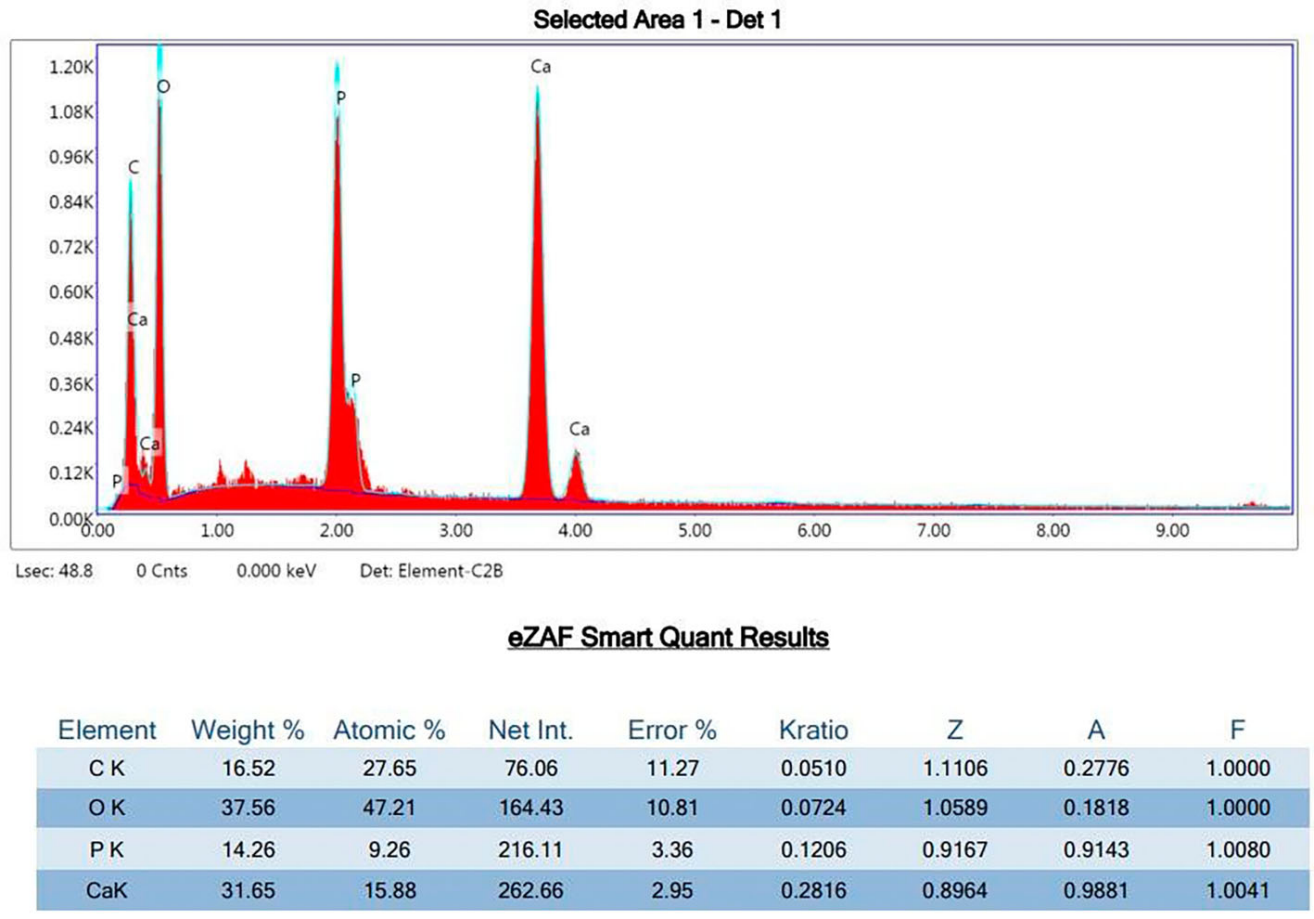
EDS spectrum of dentin showing major elements in tubular structures.

**Figure 4.**
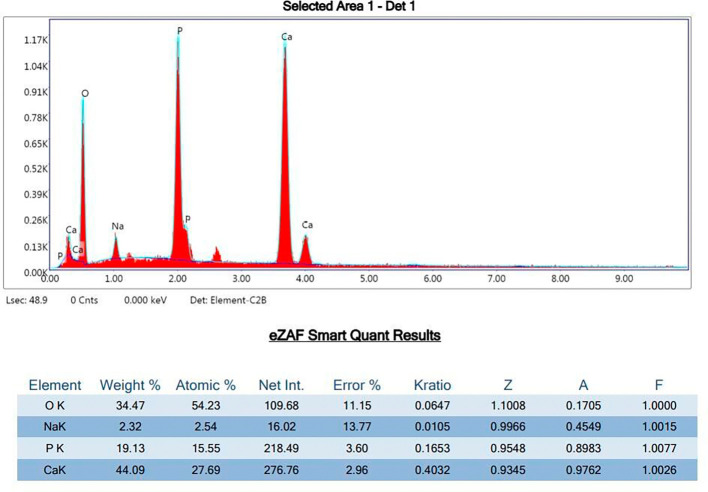
EDS quantification of mineral and trace elements within dentinal tubules.

Tubule diameters were measured using IMAGE J software. The largest diameter across each tubule was recorded to minimize error from oblique sections, with calibration based on the photomicrograph scale bar. ^17^ Measurements were converted to microns accordingly ( Figures 3 and 4).

### Grading tubule patency

*Tubule patency was graded as follows:* Grade A: Smear layer with some tubules just apparent, Grade B: Less than or equal to 10 tubules visible with majority occluded, Grade C: Greater than 10 tubules visible with majority occluded, Grade D: Less than or equal to 10 tubules visible with majority patent, Grade E: Greater than 10 tubules visible with majority patent. ^18^

### Statistical analysis

The statistical analysis was performed using SPSS v 20 (IBM Corporation, New York, USA). Kruskal Wallis H test was used to find out the difference in occlusive effects of diode laser with or without the application of nanocrystalline hydroxyapatite crystals. The significance level was fixed at p < 0.05.

## Results

The combination of the diode laser and nanocrystalline hydroxyapatite resulted in a significantly lower number of dentinal tubules (56.1 ± 22.4) compared to the diode laser alone (101.8 ± 49.9) and nanocrystalline hydroxyapatite alone (80.2 ± 27.6). The observed differences were statistically significant, with a p-value of 0.01, indicating that the combined treatment was more effective in reducing dentinal tubule density than either treatment used independently ( Table 1).

**Table 1.** Comparison of mean number of dentinal tubules between the groups.

Groups	Mean ± SD	p-value
Control	105.2 ± 19.3	0.01
Diode laser	101.8 ± 49.9	
Nanocrystalline Hydroxyapatite alone	80.2 ± 27.6	
Both Diode laser and Nanocrystalline Hydroxyapatite	56.1 ± 22.4	

The combination of the diode laser and nanocrystalline hydroxyapatite resulted in a significantly lower diameter of dentinal tubules (1.74 ± 0.23) compared to the diode laser alone (2.28 ± 0.33) and nanocrystalline hydroxyapatite alone (2.41 ± 0.35). The differences in tubule diameter among the groups were statistically significant, with a p-value of 0.00, highlighting the effectiveness of the combined treatment in reducing tubule size ( Table 2).

**Table 2.** Comparison of mean diameter of dentinal tubules between the groups.

Groups	Mean ± SD (microns)	p-value
Control	3.14 ± 0.60	0.01
Diode laser	2.28 ± 0.33	
Nanocrystalline Hydroxyapatite alone	2.41 ± 0.35	
Both Diode laser and Nanocrystalline Hydroxyapatite	1.74 ± 0.23	

## Discussion

The present study highlights the effectiveness of using a combination of diode laser and nanocrystalline hydroxyapatite for reducing dentinal tubular diameter. The use of a diode laser at 810 nm with an output power of 1 W for 60 seconds in continuous, non-contact mode seems to provide optimal conditions for sealing dentin tubules, as supported by previous findings from Umana et al. ^19^ The laser’s wavelength, which falls between 800 and 980 nm, demonstrates poor absorption in water and hydroxyapatite, allowing for deeper penetration and minimal thermal damage to surrounding dental tissues. The resultant increase in temperature effectively melts and reduces the diameter of the dentinal tubules, providing a potential solution for dentinal hypersensitivity while maintaining pulp vitality.

The combination therapy group demonstrated more substantial dentinal tubule occlusion than other groups. The findings are in line with the study conducted by Shamel et al. ^20^ The robustness of Diode Laser over hydroxyapatite nanocrystals might be related to photothermal processes, which heat and dissolve the hard tissue on the surface. ^21^ The capacity of nano-HAP to create hydroxyapatite plugs that obstruct dentinal tubules and a coating of mineral hydroxyapatite is what accounts for its efficacy in lowering DHS.

The formation of a hydroxyapatite (HAP) layer on teeth is supported by various studies indicating that nano-hydroxyapatite (nano-HAP) serves as a reservoir for calcium and phosphate ions. This helps maintain a state of supersaturation in saliva, which encourages mineral deposition on tooth surfaces. ^22, 23^ Research shows that using nano-HAP toothpaste can elevate calcium levels in saliva, enhancing this effect. ^23^ Additionally, nano-HAP can fill micropores on teeth, acting as a template for apatite deposition by attracting calcium and phosphate ions from saliva and other sources. This process promotes the integrity and growth of crystal structures within the tooth tissue. ^24^

It is widely recognized that Dentin Hypersensitivity (DHS) occurs when external stimuli lead to rapid fluid movement within exposed dentinal tubules, activating sensory nerve receptors in the pulp and causing sharp pain. Plugging these tubules with mineral hydroxyapatite (HAP) can significantly reduce their permeability, thereby minimizing fluid disturbance and alleviating DHS. ^25^ The findings of the current study align with previous research demonstrating that nano-HAP, whether in toothpaste or topical cream form, effectively reduces DHS. ^26– 32^

A key strength of our study was the use of Scanning Electron Microscopy (SEM), which offers numerous advantages such as a non-destructive approach, high resolution, three-dimensional imaging, and detailed topographical information. ^33, 34^ Additionally, various in vitro studies were conducted to analyze the mechanisms of action and desensitizing properties of the agents used. These included hydrostatic fluid filtration systems, attenuated total reflection Fourier transform infrared spectroscopy, energy dispersive X-ray analysis, confocal laser scanning microscopy, and electron spectroscopy analysis. ^35– 37^ This comprehensive methodology enhances the reliability and depth of our findings. The primary limitation of our study is the small sample size and its in vitro design. Future research should concentrate on the clinical efficacy of diode lasers with varying wavelengths, as well as the potential adverse effects of laser application on dentinal tubules. This will provide a more comprehensive understanding of the practical implications and safety of these treatments.

## Conclusion

The findings indicate that the combination of nanocrystalline hydroxyapatite powder and diode laser irradiation results in significantly better dentinal tubule occlusion compared to the diode laser alone. This suggests a synergistic effect between the two treatments, enhancing their ability to reduce dentinal tubular diameters and potentially improving the overall effectiveness in managing conditions like dentin hypersensitivity. The occlusion of open dentinal tubules is crucial for protecting the pulp from external stimuli and reducing sensitivity. The use of nanocrystalline hydroxyapatite alongside laser treatment may not only improve the structural integrity of dentin but also contribute to long-term outcomes in dental health.

### Ethical approval and consent to participate

The study was conducted in accordance with the Declaration of Helsinki and this study was approved by ethical committee of College of Dental Sciences, Davangere, with the approval number CODS/263/2020–2021. Written Informed consent was obtained from the participants for the use of their extracted teeth.

#### Consent for publication

All the participants gave consent for the publication of the data.

## Data Availability

**Figshare –** Energy Dispersive Spectroscopy of Open Dentinal Tubules on Application of Nanocrystalline Hydroxyapatite: An in vitro Study. https://doi.org/10.6084/m9.figshare.30762686.v1 ^38^ This project contains following underlying data:
• study-data.xlsx. Data are available under the terms of the Creative Commons Zero “No rights reserved” data waiver (CC BY 4.0 Public domain dedication). **Figshare** – Energy Dispersive Spectroscopy of Open Dentinal Tubules on Application of Nanocrystalline Hydroxyapatite: An in vitro Study (Informed consent form). https://doi.org/10.6084/m9.figshare.32064924.v2 ^39^ This project contains following extended data
• Informed consent form.docx Data are available under the terms of the Creative Commons Zero “No rights reserved” data waiver (CC BY 4.0 Public domain dedication).
