# Supplementary material for: Energy Dispersive Spectroscopy of Open Dentinal Tubules on Application of Nanocrystalline Hydroxyapatite: An In Vitro Study
Source: F1000Res. 2026 May 12;15:720. [Version 1] doi: 10.12688/f1000research.173988.1 (PMC13287979; doi:10.12688/f1000research.173988.1)
Supplement: Peer review history

# Reviewer report: Reviewer response for version 1

**DOI:** 10.5256/f1000research.191855.r484410

**Published:** 2026-06-22

**Authors:** Sangamesh Ningappa Chinnannavar

**Affiliations:** 1 Kalinga Institute of Dental Sciences, Odisha, India

• **Title and Abstract:**

 The title and abstract effectively summarize the key aspects of the study and provide a clear overview of its scope and objectives. However, there appears to be a discrepancy between the laser parameters reported in the Abstract and those described in the Methods section. Kindly review and correct this inconsistency.

 • **Introduction:**

 The introduction is well-structured and logically organized, supported by relevant and appropriate citations. It effectively establishes the background and rationale for the study while highlighting the importance of further research in this area.

 • **Need for the Study:**

 The rationale for the study is well justified with a sound scientific basis that emphasizes its clinical and academic significance. The study addresses an important gap in the existing literature and contributes valuable insights to the field.

 • **Methodology:**

 The methodology is clearly described and reproducible. The study design, sampling methods, and research procedures are adequately detailed. The statistical analysis performed is appropriate and robust, supporting the validity and reliability of the findings.

 • **Tables and Figures:**

 The tables and figures are relevant, well presented, and aligned with the study objectives. They effectively support the interpretation of the results. Since the Kruskal–Wallis test demonstrated statistical significance, kindly mention the post hoc test performed for multiple comparisons.

 • **Discussion:**

 The discussion is comprehensive and provides a meaningful interpretation of the study findings. The comparison with previous studies is appropriate and helps place the results within the context of existing scientific literature.

 • **Conclusion:**

 The conclusion is consistent with the study findings and appropriately summarizes the key outcomes. The inferences drawn are logical and supported by the results presented in the study.

Is the work clearly and accurately presented and does it cite the current literature?

Yes

If applicable, is the statistical analysis and its interpretation appropriate?

Yes

Are all the source data underlying the results available to ensure full reproducibility?

Yes

Is the study design appropriate and is the work technically sound?

Yes

Are the conclusions drawn adequately supported by the results?

Yes

Are sufficient details of methods and analysis provided to allow replication by others?

Yes

Reviewer Expertise:

Oral Medicine and Radiology

I confirm that I have read this submission and believe that I have an appropriate level of expertise to confirm that it is of an acceptable scientific standard.

# Reviewer report: Reviewer response for version 1

**DOI:** 10.5256/f1000research.191855.r484413

**Published:** 2026-06-15

**Authors:** Janardhana Amaranath B.J.

**Affiliations:** 1 Rama University Kanpur, Uttar Pradesh, India

The manuscript is well-structured and maintains clarity and transparency throughout the reporting. The title and abstract are informative and provide a concise overview of the study objectives, methodology, and principal findings.

 The introduction effectively establishes the need for the study by clearly outlining the research aim and objectives. The background information is relevant and adequately justifies the significance of the study within the current scientific context.

 The authors have described the validation process of the data collection instrument in detail, which significantly enhances the reliability and credibility of the study. The methodology is clearly presented and reflects careful planning and execution. The inclusion of additional details regarding the random allocation procedure, allocation concealment, and examiner blinding during SEM image acquisition and measurements would further enrich the methodological description and provide greater clarity for readers. Overall, the study is well designed, scientifically sound, and contributes valuable findings to the field, making it suitable for indexing.

 The statistical methods used are clearly specified and appear appropriate for the type of data analyzed. The results are systematically presented in well-organized tables with comprehensive and relevant information.

 The discussion provides a concise interpretation of the key findings and appropriately relates them to previous literature. Relevant studies with similar findings have been adequately cited, resulting in a balanced and informative discussion.

 The manuscript also offers useful recommendations for future research based on the study findings and acknowledged limitations. The references are appropriately formatted and adhere to the journal guidelines.

Is the work clearly and accurately presented and does it cite the current literature?

Yes

If applicable, is the statistical analysis and its interpretation appropriate?

Yes

Are all the source data underlying the results available to ensure full reproducibility?

Yes

Is the study design appropriate and is the work technically sound?

Yes

Are the conclusions drawn adequately supported by the results?

Yes

Are sufficient details of methods and analysis provided to allow replication by others?

Yes

Reviewer Expertise:

in the specialty of periodontics

I confirm that I have read this submission and believe that I have an appropriate level of expertise to confirm that it is of an acceptable scientific standard.
